# Hypothermic Machine Perfusion in Liver Transplantation—A Randomised Trial and Beyond

**DOI:** 10.3389/ti.2022.10257

**Published:** 2022-03-24

**Authors:** Peter Friend, Joerg-Matthias Pollok

**Affiliations:** ^1^ Nuffield Department of Surgical Sciences, University of Oxford, Oxford, United Kingdom; ^2^ Division of Surgery and Interventional Science, Faculty of Medical Sciences, University College London, London, United Kingdom

**Keywords:** liver transplantation, machine perfusion, hypothermic perfusion, oxygen, DCD

The randomised controlled trial of oxygenated hypothermic machine perfusion of DCD livers, reported by van Rijn et al. in NEJM in February 2021 is of great importance, not only because of the novel technology under investigation, but also because of the trial design and methodology.

After several decades in which static cold storage (SCS) has been the organ preservation methodology of choice, there is now great interest in the development of machine perfusion systems of abdominal and cardiothoracic organs. Whereas previously this was the preserve of a number of relatively simple hypothermic machine perfusion (HMP) systems for the kidney, clinical units around the world are now using/testing, perfusion systems under both cold (hypothermic) and warm (normothermic) conditions. The relatively recent demonstration of the benefits of adding oxygen to the perfusate under hypothermic conditions (1, 2) has increased the interest and application in this approach.

The publication of van Rijn et al., from the Groningen group of Robert Porte (3), addresses a vitally important question in liver transplantation: whether machine perfusion technology is of benefit with respect to the biliary complications seen in DCD transplantation. Donation after circulatory death is increasing in many countries and is now a major source of donor organs (e.g., up to 40% of all deceased donors in the United Kingdom), but utilisation of livers from DCD donors (the proportion of offered organs that result in a transplanted liver) is of the order of 25% both in Europe and the US. For this reason, the impact of DCD transplantation has been much lower in liver transplantation than in kidney transplantation. The reason for this low level of uptake is not hard to understand: not only do DCD liver transplants have a much higher rate of primary non-function and Early Allograft Dysfunction, but also there is a greatly increased risk of non-anastomotic biliary strictures (NAS), also referred to as ischaemic-type biliary lesions, and as ischaemic cholangiopathy (4). Although this complication is reported in DBD liver transplants, it is so much more commonly seen in DCD grafts as to be pathognomonic. This is the primary reason for the inferior outcomes (and higher costs) associated with DCD liver transplantation.

Within the modern era, hypothermic machine perfusion of the liver was first shown to be feasible and safe by Guarrera et al. (5), adopting relatively simple HMP technology as used in kidney transplantation. The addition of oxygenation of the circuit, pioneered by the groups at Zurich (1) and Groningen (6), later provided evidence that dissolved oxygen in an HMP circuit, even when applied for only a short period after SCS, may be associated with reduced levels of ischaemia-reperfusion injury. More is known now about the potential mechanism of benefit, much centred on the role of oxygen in maintaining aerobic mitochondrial metabolism, thereby avoiding the accumulation of succinate and subsequent release of reactive oxygen species (even at low temperature) (7). The preliminary evidence generated by the trials of hypothermic oxygenated machine perfusion (HOPE) and dual hypothermic oxygenated machine perfusion (D-HOPE) have suggested that this mitigation of ischaemia-reperfusion would translate into reduced levels of graft injury and, in particular, a reduction in the all-important syndrome of NAS. It is this hypothesis that van Rijn et al. set out to test.

The primary endpoint of this trial was the incidence of symptomatic NAS. Much has been written in recent years about endpoints for trials in organ preservation, because trials have relied upon surrogate endpoints (e.g., EAD, or peak postoperative transaminase) rather than measures of direct clinical relevance (e.g., graft survival). This is in order to design clinical trials of feasible proportion: indeed, to illustrate this, it has been pointed out that a preservation trial based upon liver graft survival would typically require in excess of 4,000 patients (8). However, the relatively high incidence of NAS in DCD liver transplants renders this complication a suitable endpoint within a trial of manageable proportions. van Rijn et al. measured, therefore, a directly clinically-relevant metric: the use of NAS as the primary endpoint in this trial is an enlightened choice.

In this trial, livers were randomised once deemed transplantable by the implanting surgical team: the trial was not, therefore, designed to test any effect on organ utilisation (a separate key issue in liver transplantation). Indeed, there was a very low discard rate of organs: only three livers were discarded as unsuitable for transplantation, these due to steatosis (*n* = 2) and retrieval damage (*n* = 1).

Similarly, the investigators did not set out to test the effect of HMP at (or beyond) the limits of current DCD practice: donors were relatively young (median age 52 years and 49 years in HMP and SCS respectively), non-obese (BMI 25 kg/m^2^ in both groups) and the warm ischaemia time were relatively short (median 11 min in both groups). Also the recipients were low-risk (MELD 14 and 16 in HMP and SCS groups respectively). Donor and patient selection was, therefore, well within the accepted range for DCD liver transplantation, and the groups were well-matched.

Diagnosis of the primary endpoint was clinical, based on the development of jaundice or cholestatic liver function tests. The diagnosis was confirmed in all cases by later MRCP. Indeed all patients underwent MRCP at 6 months postoperatively, as part of the trial protocol. This allowed not only corroboration of the clinical findings, but also objective assessment of the effect of the intervention (oxygenated HMP) with respect to biliary stricture formation. All scans were reviewed by two independent radiologists who were unaware of the treatment allocation, with a third radiologist providing the casting vote in the event of discordant opinions. The study was powered on the basis of a reduction of the NAS rate from 29% (a notably high rate in comparison to published rates) to 11%: the results of the trial showed a reduction from 18% to 6%.

The results of the protocol MRCP investigation are of some interest, because this is potentially an important endpoint in trials of future interventions in DCD liver transplantation. Here, the evidence is less clear-cut: not only did all the symptomatic patients have radiological evidence of NAS (as expected), but 70% of all MRCPs were positive, including 65% of asymptomatic patients. Including all scans graded as showing mild, moderate or severe strictures, there were no differences in the incidence or severity of radiological cholangiopathy between the two arms of the trial. As noted by the authors, this dissonance between clinical and radiological manifestations of biliary pathology is unexplained and requires more research.

Protocol MRCP assessment at 6 months postoperatively was also included in the previously-published normothermic machine perfusion trial, carried out by the Consortium for Organ Preservation in Europe (COPE): 70% of 222 transplanted patients underwent protocol MRCP. In this randomised controlled trial (9), patients receiving DCD organs comprised only a minority of the recruitment in a study with wider enrolment criteria: the incidence of radiologically-determined biliary strictures in DCD recipients was 11.1% in NMP livers (3 out of 27), compared to 26.3% in SCS livers (5 out of 19). Notably (and in line with the findings of the van Rijn paper), only two patients (one in each arm of the trial) underwent retransplantation as a result of NAS within 1 year of the initial transplant.

Other benefits were shown in the van Rijn study: these include clinically important reductions in the rate of post-reperfusion syndrome (12% vs. 27%), EAD (26% vs. 40%), the requirement for biliary interventions (5 vs. 22), and the need for readmission (6 vs. 17). These findings are all indicative of an improved preservation technology that has had the effect of reducing the severity of ischaemia-reperfusion injury. There is no doubt that such benefits are needed, especially in the context of DCD liver transplantation, in which the risk/fear of complications is responsible for organ utilisation rates of the order of 25%. However, NAS is not the only driver of poor utilisation: there are other facets of organ preservation that need improvement if optimum utilisation of the critical resource of donor organs is to be achieved.

There is little doubt that the utilisation of marginal donor organs (both DCD and DBD) is improved by the ability to assess the functional viability of the organ before deciding whether to subject a patient to the risk of transplantation. This can be achieved at normothermic temperature, potentially allowing organs that would otherwise be discarded to be transplanted: indeed a proof-of-principle study has already tested the clinical implementation of this and been published (10). Normothermic machine perfusion is intrinsically superior as a means of testing the donor organ, compared to hypothermic perfusion. As noted by van Rijn et al., there is current interest in the measurement of mitochondrial flavin mononucleotide (as a mitochondrial injury marker) during HMP, but it is not yet clear to what extent this predicts longer-term outcome (11). However, functional assessment by this or other means was not part of the study as conducted.

A further and hitherto unmet need is that of extended preservation. No real progress has been made in static cold preservation since the introduction of University of Wisconsin solution three decades ago: indeed, with more transplants of higher risk organs, average preservation times are shorter now than in the past. There is no published evidence of the utility of extending the period of safe preservation using hypothermic machine perfusion of liver grafts. Investigation of this is much needed in order to assess whether this technology is a potential solution to the very real logistic challenges of running a liver transplant programme, in which offers of donor organs may come in rapid succession, but where only one transplant can be undertaken at a time. Normothermic preservation has been shown to enable prolonged preservation times, not only allowing sequential transplantation, but also offering the real prospect of scheduling liver transplants during the day (12).

Another machine perfusion technology which is showing great promise in the context of DCD liver transplantation is that of normothermic regional perfusion (NRP)—the re-institution of oxygenated blood flow to the abdominal organs *in-situ* following the declaration of death. Although this has not been subjected to the level of randomised clinical trial analysis conducted by van Rijn et al, nonetheless accumulating evidence suggests a substantial benefit from this peri-retrieval intervention. In a publication from the United Kingdom (13), 43 livers were transplanted after NRP with no occurrence of NAS, compared with 27% in 187 DCD livers transplanted contemporaneously without the use of NRP. Notably, however, the logistics of NRP are complex, requiring additional technology and skilled personnel at the donor site, this contrasting with the much simpler logistic demands of HMP. A trial comparing HMP, NMP, and NRP in the management of DCD livers is much needed.

After several decades of relative stagnation, the field of transplant organ preservation is undergoing a renaissance, with the implementation of machine perfusion systems. Although it is easy to characterise the current state of the art as a debate about hypothermic vs. normothermic perfusion, it is likely that future implementations will exploit temperature not as a binary but as a continuous variable parameter with temperature transition being seen as a key issue. Also, not only will organs be thereby preserved in better condition and for longer, but specific targeted interventions will be applied to repair and modify organs to the benefit of post-transplant outcomes. The delivery of oxygen at cold temperatures is just a first step into this exciting future.
